# Interment and Disinterment; or a Further Exposition of the Practices Pursued in the Metropolitian Places of Sepulture, as Affecting the Health of the Living

**Published:** 1843-04

**Authors:** 


ART. XII.—*Interment and Disinterment; or a Further Exposition of the Practices pursued in the Metropolitan Places of Sepulture, as affecting the Health of the Living.* By S. A. WALKER, Surgeon.—*London,* 1843. 8vo, pp. 28.

THIS is a very important addition to the author's work on Graveyards; and we regret much not having seen it until our article on Cemeteries in the present Number was printed. Mr. Walker's pamphlet contains many new facts, strikingly illustrative of the evils flowing from the barbarous practice of intra-mural sepulture, evils which, thanks to the author and his patriotic coadjutors, are now felt to be so intolerable as to command abatement. In reference to this question, ignorance and self-interestedness may continue to be blind and deaf; but we shall be content to abide by the verdict of all honest and unprejudiced men who shall read Mr. Walker's pamphlet. To such we commend it.

